# Robust Detection of Somatic Mosaicism and Repeat Interruptions by Long-Read Targeted Sequencing in Myotonic Dystrophy Type 1

**DOI:** 10.3390/ijms22052616

**Published:** 2021-03-05

**Authors:** Antoine Mangin, Laure de Pontual, Yu-Chih Tsai, Laetitia Monteil, Mathilde Nizon, Pierre Boisseau, Sandra Mercier, Janet Ziegle, John Harting, Cheryl Heiner, Geneviève Gourdon, Stéphanie Tomé

**Affiliations:** 1 Centre de Recherche en Myologie, Inserm, Institut de Myologie, Sorbonne Université, F-75013 Paris, France; mangina@cardiff.ac.uk (A.M.); l.depontual@institut-myologie.org (L.d.P.); genevieve.gourdon@inserm.fr (G.G.); 2 Dementia Research Institute, Cardiff University, Cardiff CF10 3AT, UK; 3 Pacific Biosciences, Menlo Park, CA 94025, USA; ytsai@pacificbiosciences.com (Y.-C.T.); jziegle@pacificbiosciences.com (J.Z.); jharting@pacificbiosciences.com (J.H.); cheiner@pacificbiosciences.com (C.H.); 4 Genetics Department of the Hospital of Toulouse, F-31059 Toulouse, France; monteil.l@chu-toulouse.fr; 5 CHU de Nantes, Service de Génétique Médicale, Laboratoire de Génétique Moléculaire, F-44000 Nantes, France; mathilde.nizon@chu-nantes.fr (M.N.); pierre.boisseau@chu-nantes.fr (P.B.); 6 CHU Nantes, Service de Génétique Médicale, Centre de Référence des Maladies Neuromusculaires AOC, F-44000 Nantes, France; Sandra.MERCIER@chu-nantes.fr

**Keywords:** myotonic dystrophy type 1, long read sequencing, somatic mosaicism, interrupted CTG repeat expansion

## Abstract

Myotonic dystrophy type 1 (DM1) is the most complex and variable trinucleotide repeat disorder caused by an unstable CTG repeat expansion, reaching up to 4000 CTG in the most severe cases. The genetic and clinical variability of DM1 depend on the sex and age of the transmitting parent, but also on the CTG repeat number, presence of repeat interruptions and/or on the degree of somatic instability. Currently, it is difficult to simultaneously and accurately determine these contributing factors in DM1 patients due to the limitations of gold standard methods used in molecular diagnostics and research laboratories. Our study showed the efficiency of the latest PacBio long-read sequencing technology to sequence large CTG trinucleotides, detect multiple and single repeat interruptions and estimate the levels of somatic mosaicism in DM1 patients carrying complex CTG repeat expansions inaccessible to most methods. Using this innovative approach, we revealed the existence of de novo CCG interruptions associated with CTG stabilization/contraction across generations in a new DM1 family. We also demonstrated that our method is suitable to sequence the DM1 locus and measure somatic mosaicism in DM1 families carrying more than 1000 pure CTG repeats. Better characterization of expanded alleles in DM1 patients can significantly improve prognosis and genetic counseling, not only in DM1 but also for other tandem DNA repeat disorders.

## 1. Introduction

More than 40 different human disorders are caused by tri-, tetra-, penta- or hexanucleotide repeat expansions localized either in coding or non-coding regions of the target gene [1]. The pathogenic mechanisms for repeat diseases involve either a loss of protein function or a gain of function at the RNA or protein level, depending on the type and location of the repeat [1]. Among the trinucleotide repeat (TNR) diseases, Fragile X syndrome (FXS (MIM: 300624)), Huntington’s disease (HD (MIM: 143100)), several spinocerebellar ataxias (SCAs) and myotonic dystrophy type 1 (DM1 (MIM: 160900)) have been reported. DM1 is a highly multisystemic disorder caused by an unstable CTG repeat expansion within the 3′-untranslated region (UTR) of the myotonic dystrophy protein kinase (*DMPK*) gene that usually increases across generations and in tissues [2,3]. DM1 is mainly characterized by a broad clinical spectrum of symptoms such as myotonia, muscle weakness, cardiac conduction defect, respiratory insufficiency, dysphagia, gastrointestinal symptoms, somnolence or cataracts [4]. Several parameters such as the CTG repeat length and gender contribute to the phenotypic variability of DM1, resulting in five distinct clinical forms from late onset to the most congenital cases, which are often associated with the largest size of inherited disease-associated allele [4,5]. Facial dysmorphisms, muscle weakness and cognitive impairment are more frequent symptoms in earlier onset form while cardiac defects and cataracts are seen more in DM1 patients with later forms of the disease. Interestingly, gastrointestinal problems or dysphagia and insomnia are found in all five forms of DM1 described in a large French DM1 cohort [4]. However, many DM1 patients develop unusual DM1 symptoms or remain asymptomatic despite the presence of a large CTG repeat expansion in their cells. This suggests that contributing factors such as somatic mosaicism, gene modifiers and environmental factors may affect the evolution of the clinical and mutation aspects [3,6,7]. The somatic mosaicism observed in blood is strongly biased towards expansions and contributes not only to the progressive nature of the different symptoms in several DM1 ethnic groups, but also to the variation in the age of onset [6,8,9,10]. Two studies also revealed that single-nucleotide polymorphisms in the MutS Homolog 3 (*MSH3*) DNA mismatch repair gene may reduce somatic mosaicism levels but also delay onset in DM1 patients [11,12].

More recently, interrupted TNR alleles have been associated with stabilization of TNR repeat loci and a change in clinical features in DM1 as well as in other TNR diseases such as fragile X-associated tremor/ataxia syndrome (FXTAS (MIM: 300623)) or fragile X-associated primary ovarian insufficiency and SCA1 (MIM: 164400) [13,14,15,16,17,18,19,20,21,22,23]. Several studies have described various types of interruptions in the 5′ and 3′ ends of the CTG expanded allele in 3–8% of the DM1 population [16,17,18,19,20,21,22,23,24,25,26,27,28,29,30,31,32]. These interruptions are associated with stabilization of CTG repeat expansion and atypical clinical features. Most CCG interruptions are often associated with milder DM1 symptoms and/or additional symptoms [17,18,19,21,23]. Interrupted CTG repeat sequences have been associated with late onset of symptoms and severe atypical axial and proximal weakness. No milder symptoms have been described in Spanish DM1 patients with interrupted CTG repeat expansion [21]. Compared to the TNR-coding diseases and FXS, the type, number and position of interruptions are extremely variable in DM1. The disparity of interruptions may explain the broad clinical spectrum observed in patients carrying interrupted sequences and may also make it difficult to understand the role of these interruptions on the phenotype.

Accurate estimation of the size of inherited CTG repeat expansion, somatic mosaicism and identification of interrupted alleles are crucial to better characterize the genotype–phenotype correlation in DM1. Inherited CTG repeat expansion size and the level of somatic mosaicism are traditionally evaluated by Southern blot, polymerase chain reaction (PCR) and small pool PCR [33]. These methods do not provide any information on the sequence of CTG repeat expansion. Only triplet-primed PCR may detect the presence of interruptions at the 5′ and 3′ ends of the CTG repeat expansion [34]. Identification of interruptions may be resolved by short-read sequencing or by enzymatic digestion [17,34]. However, the information obtained is limited to the end of the sequence and gives no information about the middle of the sequence.

Long-read sequencing has recently been successfully applied in HD and Fragile X patients and also in DM1 patients [18,35,36,37,38]. The Monckton group analyzed the CTG repeat expansions in different DM1 patients with less than 400 CTG repeats using a new long-read technology, single molecule real-time (SMRT) sequencing by Pacific Biosciences (PacBio), and the penultimate PacBio RSII System [18]. They showed that CCG interruptions are exclusively localized at the ends of the sequence and are associated with milder symptoms in DM1 patients with <400 CTG repeat tracts. Today, no method has been described to study both the size of large CTG repeats, the presence of interruptions and somatic mosaicism in DM1.

The present study is an extension of previously reported results in DM1 patients using the latest generation of long-read sequencing developed by PacBio and amplicons as resources. We have shown that the new PacBio technology can sequence at least 1000 CTG repeats, detect a single CAG and multiple CCG interruptions and estimate somatic mosaicism at the same time with sufficient depth. In this study, we described a paternal de novo CCG interruption in a new DM1 family. We also characterized the CTG repeat sequence and its haplotype in seven individuals of a large family (three generations) carrying a DM1 intermediate allele (37 repeats). We have identified the stable interrupted hexamer allele (CCGCTG) associated with the European DM1 haplotype A. More complete characterization of the expanded allele in DM1 patients will improve our knowledge on the genotype–phenotype correlation in DM1 as well as the prognosis and genetic counseling in this disease and other TNR disorders.

## 2. Results

### 2.1. De Novo CCG Interruption in New DM1 Family Identified with the Sequel II System

**Figure 1:**
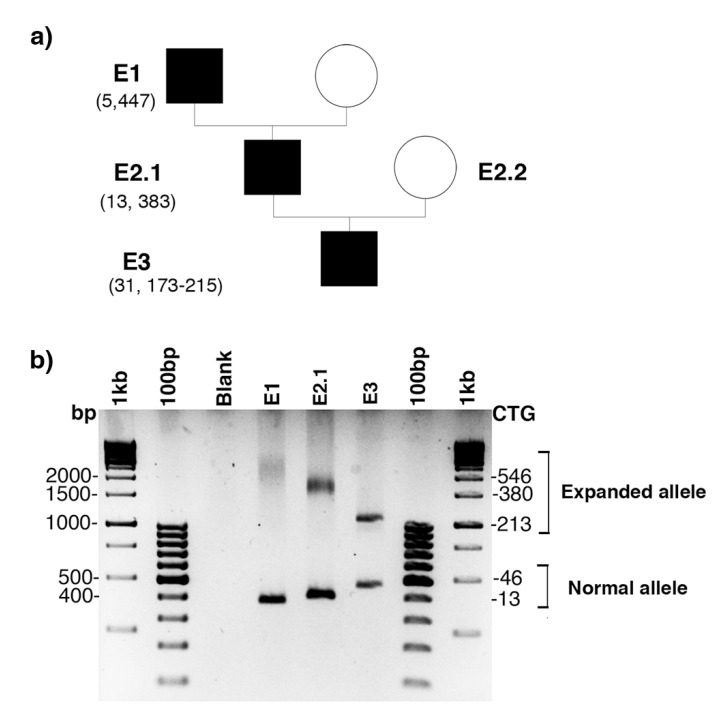
Atypical myotonic dystrophy type 1 (DM1) family E. (**a**) Part of family E pedigree. CTG repeat length estimated by Sequel II System is placed in parentheses. (**b**) PCR amplification of the CTG repeats. Normal and expanded alleles are indicated in brackets. The sizes of DNA molecular weight 1 kb (Thermo Fisher Scientific, Courtaboeuf, France) in base pairs and in the number of CTG repeats are shown on the left and the right sides of the figure, respectively.

**Figure 2:**
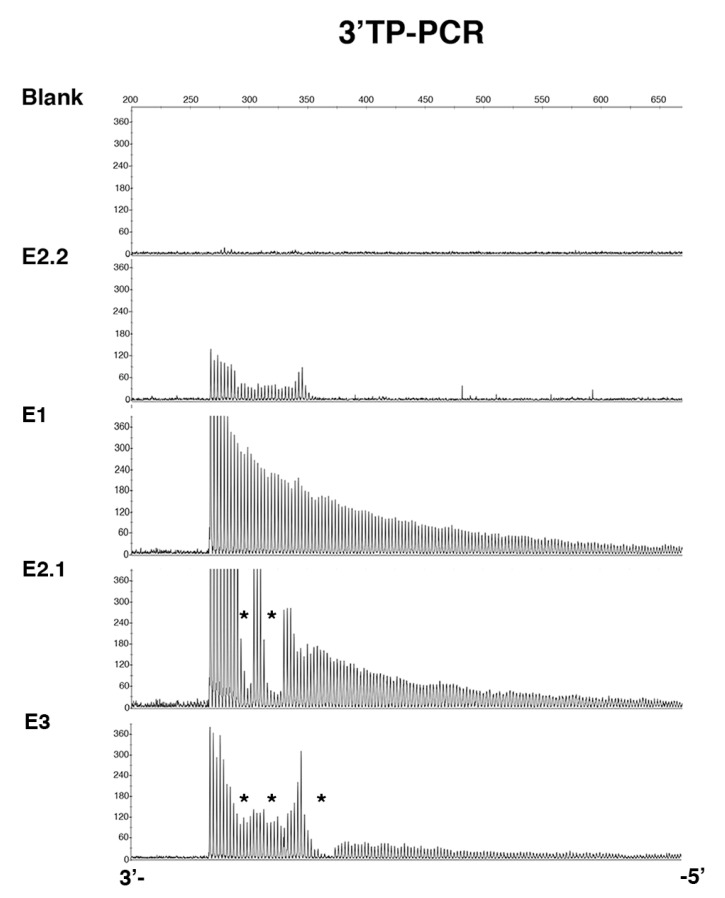
3′ triplet-primed polymerase chain reaction (TP-PCR) results in individuals from family E. The *y*-axis represents the intensity of fluorescence (arbitrary unit) and the *x*-axis represents the fragment length in base pair. An asterisk (*) indicates the localization of the interruptions.

**Figure 3:**
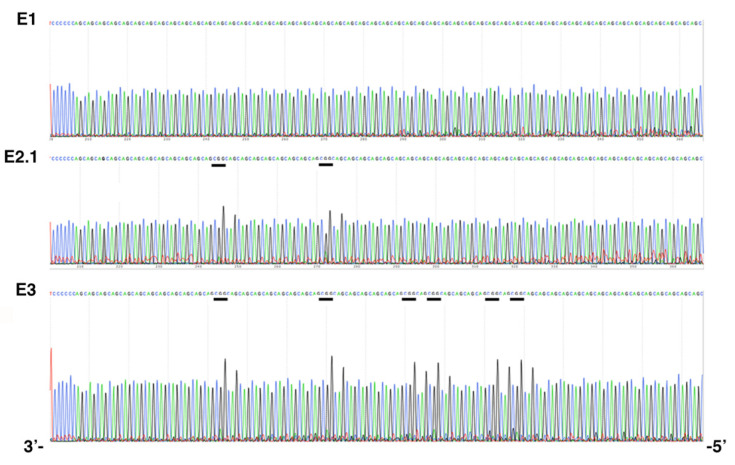
DM1 locus sequences in the family E (cloning sequencing). GGC (CCG) interruptions are underlined.

**Figure 4:**
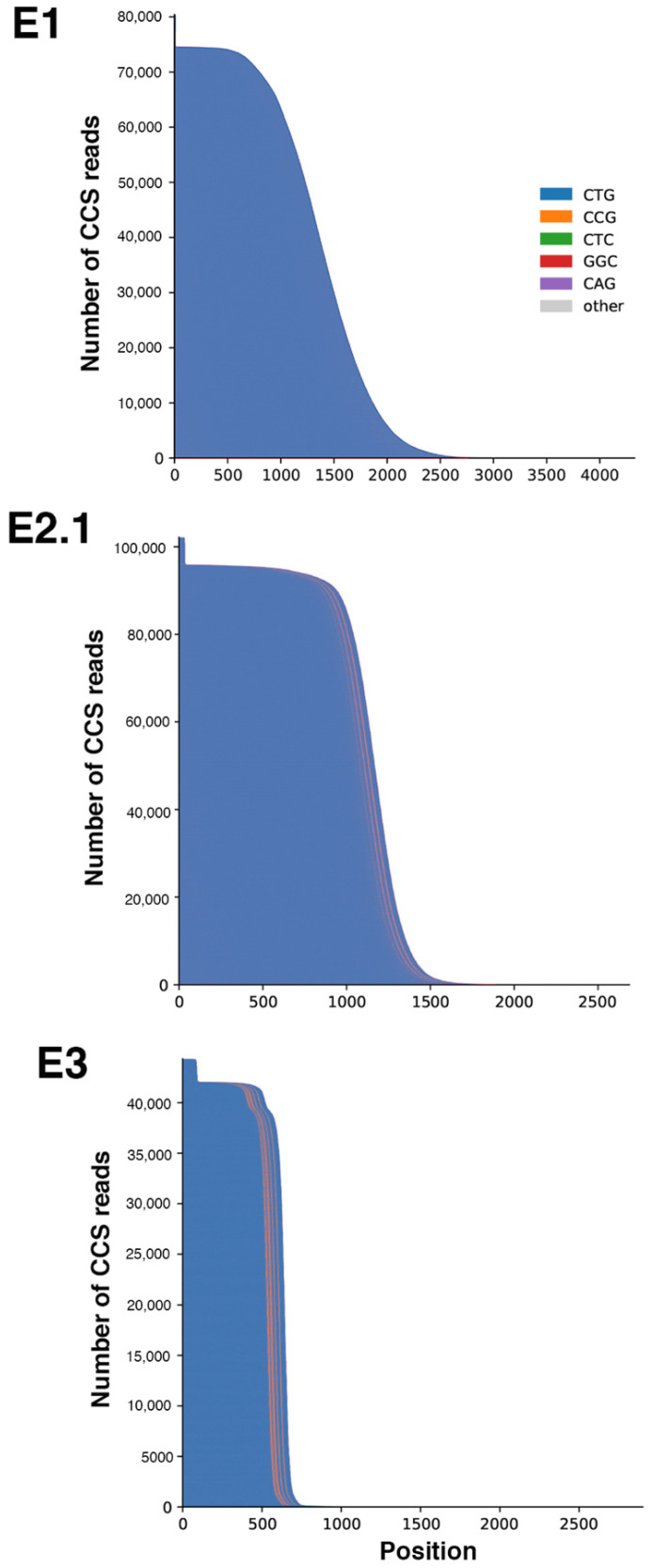
Waterfall plots outline the repeat structure of the normal and expanded alleles in family E. The *y*-axis shows the number of circular consensus sequencing (CCS) reads whereas the *x*-axis shows the length of the CTG repeat expansion in base pairs. The CTG repeat is represented in blue whereas the CCG interruptions are represented in orange. The highest peaks at the far left of the distribution represent the normal allele.

Family E was recruited for prenatal genetic counseling. E2.1 was identified as a DM1 patient carrying interrupted CTG repeat expansion with atypical symptoms. In order to improve the genetic counseling in this family, we mainly characterized the *DMPK* mutation in DM1 members of this family. PCR amplification of the CTG repeat tracts revealed a decrease in CTG repeat size across two paternal transmissions (Figure 1). Bidirectional triplet-primed polymerase chain reaction (TP-PCR) showed several interruptions at the 3′ end of the CTG repeat in individuals E2.1 and E3. However, the TP-PCR trace is different between E2.1 and E.3 (Figure 2). Two distinct gaps were found in both TP-PCR traces, whereas a third gap was only observed in individual E.3, introducing the presence of additional interruptions in the fetus E3. Interestingly, the 3′ end of the repeat appeared free of interruption in individual E.1 (Figure 2). By cloning and sequencing the CTG repeat tracts, we identified a majority of expanded *DMPK* alleles with two and six non-consecutive CCG interruptions in the 3′ of the CTG expansion of E2.1 and E3, respectively, whereas no interruption was identified in individual E1 in the ends of the CTG repeat expansion (Figure 3). These first results suggest de novo mutation in this family. However, the TP-PCR method and cloning sequencing did not allow the characterization of the middle of the expanded repeats in this family, particularly in individual E1 with the largest expanded allele. In order to definitively exclude the presence of interruption in the unsequenced CTG repeat region by conventional methods in E1, we analyzed around 10,000 molecules of *DMPK* alleles from the blood of family E using SMRT sequencing on the Sequel II System. First, we sequenced the full repeat expansion for each patient and accurately estimated the size of the CTG expansion. The mode of the CTG repeat size frequency distribution was 447, 383 and 173/215 CTG repeats in E1, E2.1 and E3 individuals, respectively (Table 1). In the E3 fetus, the CTG repeat length distribution was bimodal with a mode at ~173 and ~215 compared to the individuals E1 and E2 which show a unimodal distribution. We identified CCG interrupted alleles exclusively in individuals E2.1 and E3 whereas the individual E1 carried a pure expanded allele (Figure 4 and Table 1). Interestingly, the SMRT sequencing revealed cells carrying a majority of expanded *DMPK* alleles with two or three CCG interruptions in individual E2.1, suggesting that the number of interruptions varies between cells in blood (Table 1 and data not shown). The results of SMRT sequencing confirm that de novo interruptions occur during the E1 and E2.1 paternal transmission.

### 2.2. Sequel II System Makes It Possible to Estimate the CTG Repeat Length and the Interruptions as Well as the Somatic Mosaicism in DM1 Patients with at Least 1000 CTG Repeats

**Figure 5:**
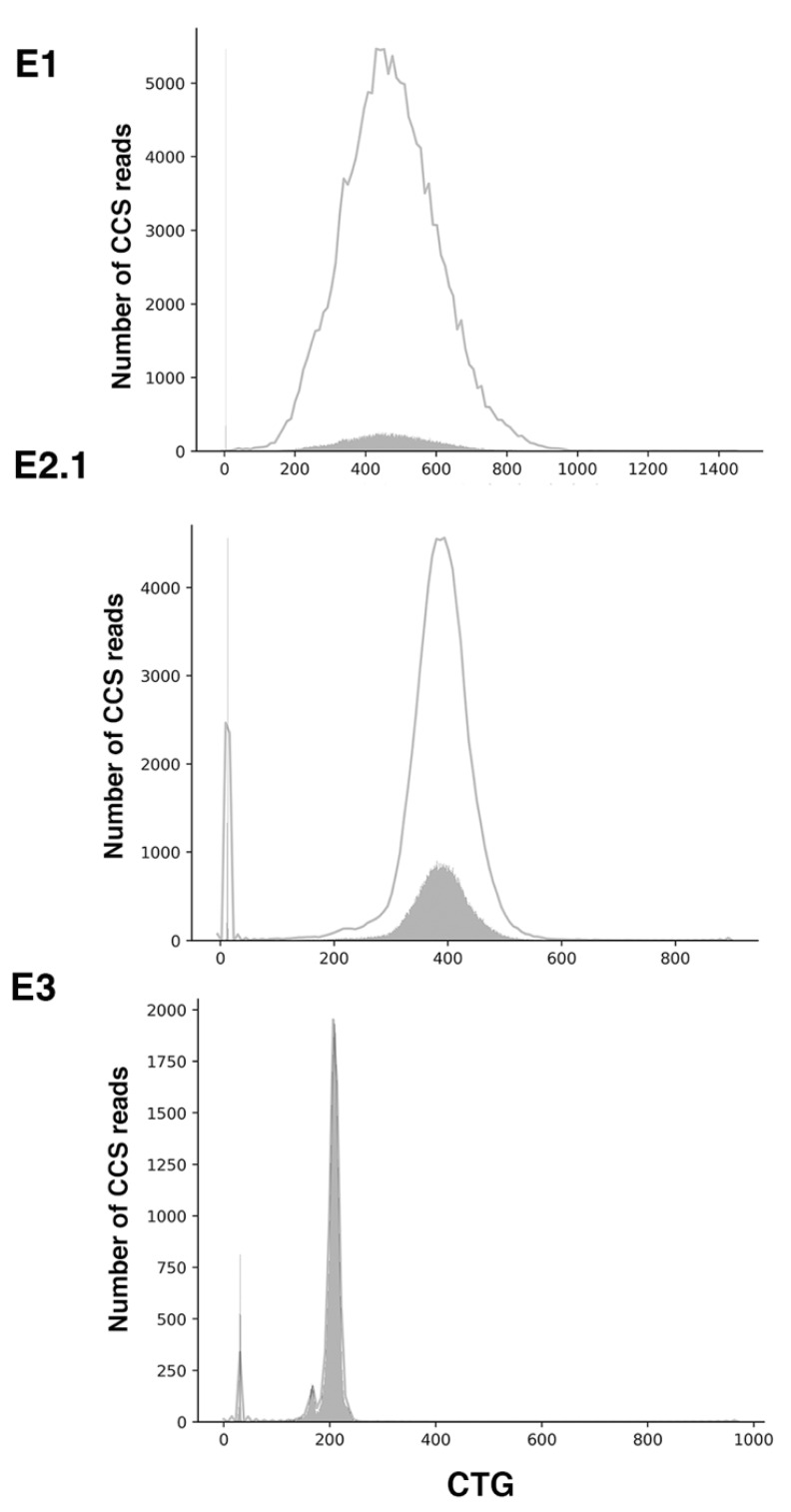
CTG repeat size distribution in family E. The *y*-axis shows the number of CSS reads in the solid grey distribution, whereas the *x*-axis shows the number of CTG repeats. Repeat counting allows to confirm allele sizes for both normal and expanded alleles as well as the extent of mosaicism of the expanded allele. The grey line represents a kernel density estimation of the underlying solid grey distribution of CCS reads.

**Figure 6:**
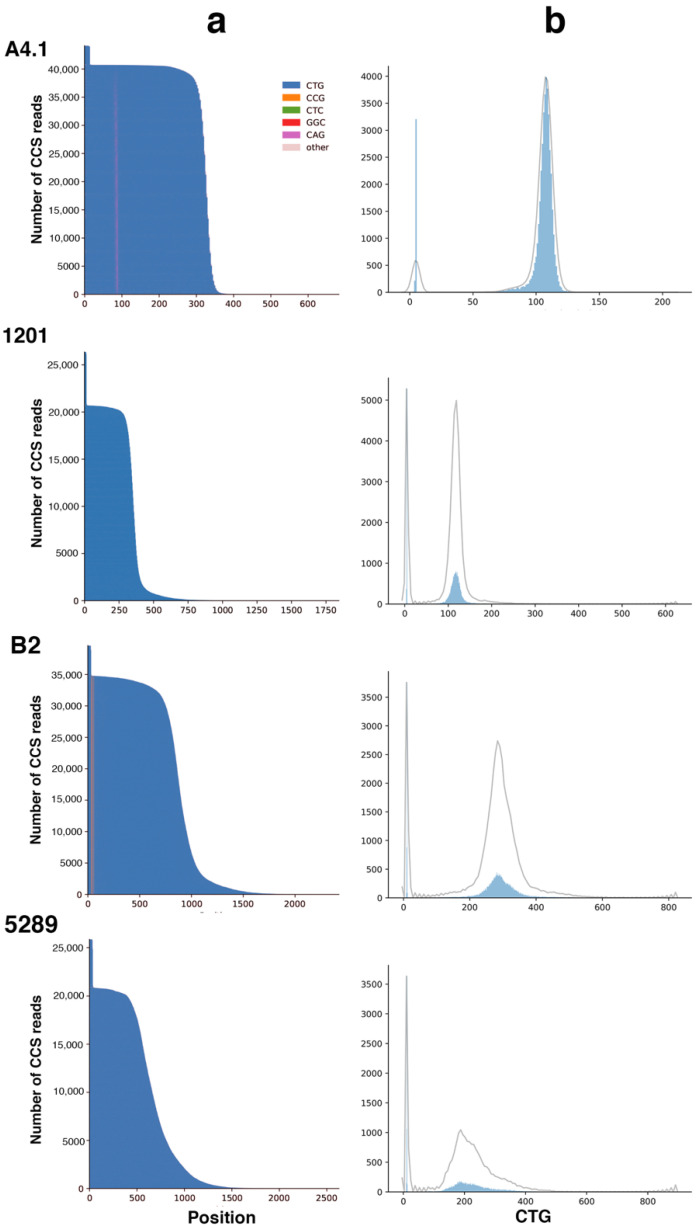
Long-read sequencing results in four DM1 patients (**a**)—Waterfall plots outline the repeat structure of the normal and expanded alleles. The *y*-axis shows the number of CSS reads whereas the *x*-axis shows the length of the CTG repeat expansion in base pairs. The CTG repeat is represented in blue whereas the CAG interruption is represented in purple and the CCG interruptions are represented in orange. The highest peaks represent the normal allele. (**b**)—CTG repeat size distribution. The *y*-axis shows the number of CSS reads in the solid blue distribution, whereas the *x*-axis shows the number of CTG repeats. The grey line represents a kernel density estimation of the underlying blue distribution of CCS reads. A4.1 (CAG interrupted expansion), B2 (CCG interrupted expansion), 1201 and 5289 carry pure CTG repeat expansions.

**Figure 7:**
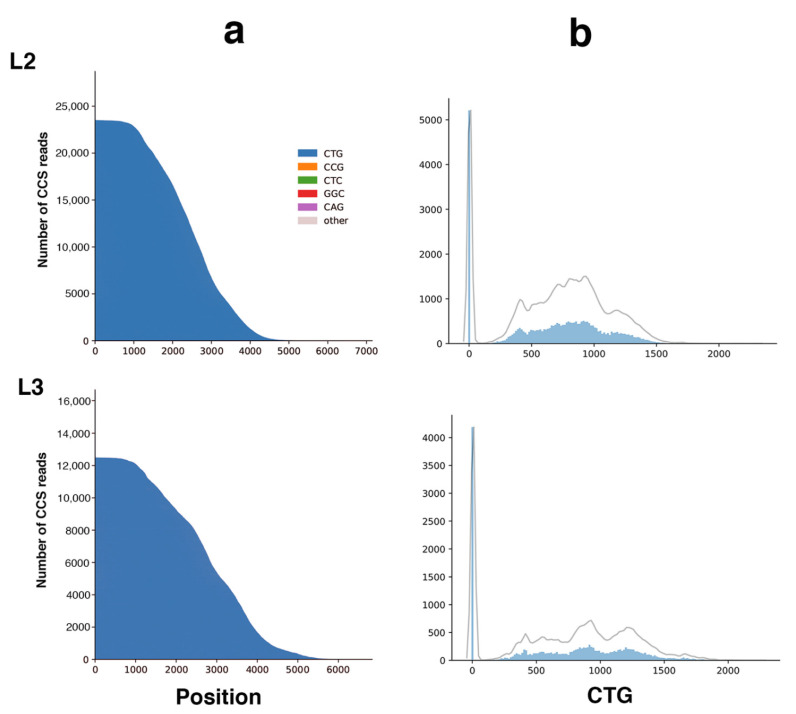
Long-read sequencing results in individuals L2 and L3 carrying more than 1000 CTG repeats. (**a**)—Waterfall plots outline the repeat structure of the normal and expanded alleles. The *y*-axis shows the number of CSS reads, whereas the *x*-axis shows the length of the CTG repeat expansion in base pairs. The CTG repeat is represented in blue. The highest peaks at the far left of the distribution represent the normal allele. (**b**)—CTG repeat size distribution in DM1 patients with more than 1000 CTG repeats estimated at diagnosis. The *y*-axis shows the number of CSS reads in the solid blue distribution, whereas the *x*-axis shows the length of the CTG repeat expansion. The grey line represents a kernel density estimation of the underlying blue distribution of CCS reads.

Using data generated by the Sequel II System, we were able to sequence CTG repeat expanded allele in individuals E1, E2.1 and E3 and then accurately estimate the size of the repeat and the number of interruptions. Interestingly, SMRT sequencing also allowed for quantifying the degree of somatic mosaicism in these DM1 patients. The level of somatic mosaicism is higher in individuals E1 than E3 (Figure 5). To support the efficiency of SMRT sequencing in DM1 and to strengthen our data, we also analyzed the CTG repeat expansions in patients A4.1 (single CAG interruption) and B2 (3 CCG interruption) and two DM1 patients (1201 and 5289) with pure CTG repeat expansions published in Tomé et al. (Table 1 and [16]). As previously described, we identified a single CAG repeat interruption in individual A4.1 and three CCG interruptions in individual B2 (Figure 6a and Table 1). In addition, somatic mosaicism was observed in four DM1 patients, with lower somatic mosaicism in individuals A4.1 and B2 compared to the respective DM1 patients with pure repeats as reported in Tomé et al. (Figure 6b). For the first time, we also succeeded in sequencing the DM1 locus and estimated somatic mosaicism in the DM1 family (patients L2 and L3) carrying more than 1000 pure CTG repeats using the Sequel II System (Figure 7 and Table 1). The mode of the CTG repeat size frequency distribution was 957 and 1156 repeats in individuals L2 and L3, respectively (Table 1). No interruption was identified (Figure 7a) and a high somatic mosaicism was observed in these two patients where the largest expanded allele contained 2138 CTG repeats (Figure 7b). We have shown that the Sequel II System successfully analyzes the sequence of CTG repeats in DM1 patients carrying more than 1000 CTG repeats, the degree of somatic mosaicism and the variant in a single analysis.

### 2.3. Stable CCG-Interrupted Allele with 37 Repeats Is Associated to DM1 Haplotype in a Large Family

**Figure 8:**
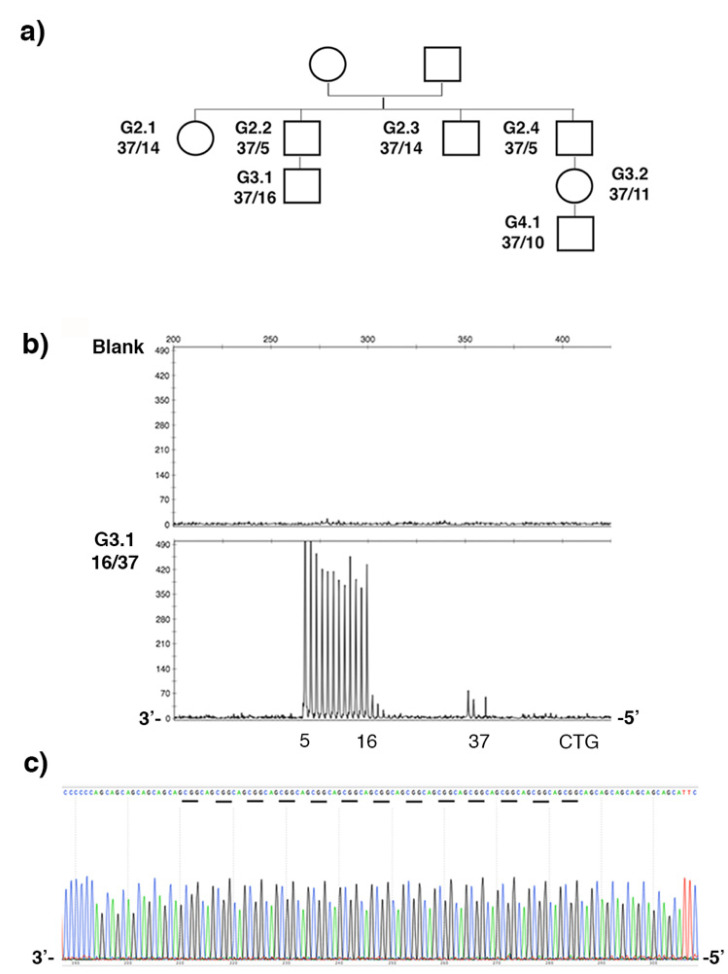
G37 family. (**a**) Large family carrying DM1 intermediate allele. The CTG repeat length of normal and intermediate alleles are indicated. (**b**) The 3′ TP-PCR results in a blank and the individual G3.1. The *y*-axis represents the intensity of fluorescence (arbitrary unit) and the *x*-axis represents the fragment length in base pairs. (**c**) Sequence result in individual G3.1. The CCG interruptions are highlighted.

DM1 disease was suspected during maternal and fetal monitoring in the individual G3.2 from the G37 family (Figure 8a). By classic PCR and Sanger sequencing, we identified a 37-CTG repeat allele in the individuals G3.1 and G3.2 as well as in the other members of the family, excluding the presence of DM1 in this family (Figure 8). This allele is stably transmitted across successive generations as expected. In order to better characterize the nature of the CTG repeat locus in this family, we utilized TP-PCR at the 3′ ends of the CTG repeat in blood samples from different members of the G37 family. The 3′ TP-PCR experiment revealed an unexpected pattern of the electrophoretic peak with a large gap, suggesting the presence of several interruptions in the largest CTG repeat allele (Figure 8b). By direct sequencing, we identified (CCGCTG) hexamer interruptions in the repeat. All members of this family carry a stable allele 5′-(CTG)_6_(CCGCTG)_13_(CTG)_5_-3′ (Figure 8c and data not shown). In order to understand the origin of interruption in DM1 disease, we genotyped different polymorphic markers in the DM1 locus. First, we showed that a (CTG)_6_(CCGCTG)_13_(CTG)_5_ interrupted allele is associated with the *Alu* insertion polymorphism in the G37 family (Table 2 and data not shown). We completed our haplotype analysis by genotyping other polymorphisms in the DM1 locus (Table 2). Our results showed that the interrupted 37 CTG repeat alleles are associated with haplotype A and are shared by the majority of pure and interrupted DM1 alleles [16,17,29,39,40,41].

## 3. Discussion

Genetic counseling for DM1 is very complex due to the highly variable clinical presentation and technical difficulties in determining the size and variant repeat interruptions of the large CTG repeat expansions. For several years, the size of the repeat expansions, the degree of somatic mosaicism and *DMPK*-interrupted alleles have been established as genetic modifiers of DM1 symptoms [3]. A decrease in somatic mosaicism and CTG repeat length is usually associated with a decrease in the severity and age of onset of DM1 symptoms [6,8]. The interruptions are associated with a stabilization of the repeat and a modification in the progression of the DM1 symptoms [16,17,18,19,21,22,23,32]. It is, therefore, crucial to develop a simple and rapid analysis of large CTG repeat expanded sequences to significantly improve our knowledge of DM1. Recently, another study analyzed CTG repeat expansions in different DM1 patients using the penultimate PacBio RSII System. They showed that CCG interruptions are exclusively localized at the end of the sequence in DM1 patients with less than 400 repeats [18]. However, no data were obtained in DM1 patients with larger repeats. Here, we have analyzed, for the first time, two DM1 families carrying CTG repeats ranging from 170 to over 1000 CTG repeats using the Sequel II System. The Sequel II System generates longer reads, enabling higher CCS accuracy, and has higher throughputs than the Sequel and RSII Systems (data not shown and [18,42,43]). Here, the Sequel II System and bioinformatic tools give us the ability to simultaneously measure repeat numbers with high resolution, to resolve the complete sequence complex repeat expansions and to measure the degree of somatic mosaicism. We have shown that the Sequel II System allows for sequencing a large repeat expansion as large as 2000 CTG repeats in DM1 patients of the family L with more accuracy than conventional PCR (data not shown). In the family E, we identified a major allele with two de novo CCG interruptions occurring across the E1 and E2.1 paternal transmission by the Sequel II System. Interestingly, the number of interruptions increased from one generation to the next in family E, as previously described in several analyses (Table 3). Using the Sequel II System, we have reported that CCG interruptions at the 3′ end of CTG repeat expansions are associated with CTG stabilization/contraction across generations. Here, the Sequel II System makes it possible to estimate the frequency distribution of the CTG repeat. The level of somatic mosaicism is the highest in L2 and L3 with the largest repeat. In family E, the degree of somatic mosaicism is higher in E1 than in E3, suggesting an age- and size-dependent effect on the somatic mosaicism of the DM1 locus. Strikingly, our new data are consistent with previous studies showing that the dynamics of CTG repeat instability are altered by repeat interruptions and the size and age of DM1 patients using conventional PCR or small pool PCR [10,16,17,22,44,45,46]. The E3 fetus exhibits two major CTG repeat lengths with approximately 170 and 215 CTG repeats, suggesting that the CTG repeat instability is already detectable at the early stage of embryogenesis as previously reported in the literature [47,48].

The Sequel II System successfully sequences CTG repeat expansions in our DM1 families. This new technology is a straightforward way to detect clinically significant repeat changes and estimate the size of the repeat in blood using targeted sequencing with PacBio SMRT sequencing. Despite the advanced PacBio technology, amplicon-based long-read sequencing still depends on PCR and the inherent bias towards preferential amplification of smaller repeats. To overcome this limitation, amplification-free targeted sequencing has been first described in a Fuchs’ endothelial corneal dystrophy-associated Transcription Factor 4 (TCF4) CTG triplet repeat [49]. The procedure consists of sequencing targeted genomic regions, without amplification, on a PacBio System by using Clustered Regularly Interspaced Short Palindromic Repeat (CRISPR)-Cas9 enrichment technology [38]. This approach should improve the analysis of CTG repeat expansions and somatic mosaicism in DM1 and also in other TNR diseases.

As noted above, we have identified family E with de novo CCG interruptions at the 3′ end in the CTG repeat expansion (Table 1). The percentage of interrupted expanded alleles has been estimated at 3–8% in the non-African DM1 population (Table 3 and [16,17,18,19,20,22,24,25,26,27,28,29,30,31,32]). To date, the origin of the interruptions remains very obscure. In our study, we reported a normal interrupted allele with 37 stable CTG repeats (5′-(CTG)_6_(CCGCTG)_13_(CTG)_5_-3′) associated with haplotype A in a large French family (two paternal transmissions and one maternal transmission). Our data suggest that the size and interruption pattern of this allele remain stable through generations. Two other analyses showed that stable CCG-interrupted 37 or 41 repeat alleles share a common haplotype A with DM1 mutation [17,29]. The DM1 haplotype A was also found in DM1 patients with interrupted expanded alleles, suggesting that the normal allele might be a source of imperfect expanded alleles found in less than 10% of DM1 patients [16,17,29,39,40,41]. However, the profile/type of interrupted alleles found in patients carrying 37, 38, 41, 43 or more than 50 repeats is extremely variable within DM1 families and also between DM1 families (Table 3), which does not suggest any haplotype specificity of the interrupted alleles. In addition, stabilization of a repeat by interruptions does not favor the hypothesis of an interrupted normal allele as the source of the interrupted expanded allele in the DM1 population [10,16,17,22,31]. The heterogeneity of the number and type of interruptions observed in the interrupted expanded alleles suggests new mechanisms leading to base substitution in the sequence and/or duplication of existing interruptions in the repeated sequence (Table 3). The emergence of interruptions can be caused by multiple processes including spontaneous DNA damage, DNA repair and DNA polymerase errors occurring in germ cells and somatic cells throughout embryogenesis and the lifetime of DM1 patients.

To conclude, we used the latest generation of the long-read sequencing system in DM1 patients with more than 1000 CTG repeats that allows detection of a single nucleotide change in the sequence and estimates the size of the large repeated sequence and somatic mosaicism at the same time. SMRT sequencing opens new avenues for DM1 disease and will provide a better understanding of the clinical and genetic variability observed in DM1 through global analysis. Growth in users of SMRT sequencing and reduction in its price will enable SMRT sequencing to be implemented as a routine molecular diagnostic method offering the best diagnostics and prognosis for patients in the near future. Our study reinforced the idea that interrupted alleles do not originate from an ancestral/normal allele but from unknown mechanisms occurring both in the germline and in somatic cells.

## 4. Materials and Methods

### 4.1. Patient Recruitment

Individuals from family G37 and DM1 patients were recruited by the Genetics Department of the Hospital of Nantes, the Genetics Department of the Hospital of Toulouse, the Genetics Department of the Necker-Enfants Malades Hospital and the DM-Scope registry [50] in France. Major clinical data are available for DM1 patients from family E. Each patient gave informed consent stating that their DNA samples could be used for research purposes. The individuals A4.1, 1201, B2 and 5289 described in Tomé et al. and analyzed by SMRT sequencing in this study are not related to each other [16].

### 4.2. CTG Repeat Amplification

To precisely estimate the inherited CTG repeat length in all individuals, 5 ng of DNA from blood or trophoblast was amplified in a 25-µL reaction using 0.4 µM ST300F (5′-GAACTGTCTTCGACTCCGGG-3′) and ST300R (5′-GCACTTTGCGAACCAACGAT-3′) primers, 1× Custom master mix (Thermo Fisher Scientific, Courtaboeuf, France) and 0.04U Thermoperfect *Taq* polymerase (Peak International Products b.v, LZ Eerbeek, Netherlands). The following cycling conditions were used: 5 min at 96 °C; 45 s at 96 °C, 30 s at 60 °C and 3 min at 72 °C (30 cycles); 1 min at 60 °C and 10 min at 72 °C (1 cycle). PCR product was mixed with orange DNA loading dye and run on a 1.5% agarose gel at 120 V. The size of the PCR products was measured using Bio-Rad’s Image Lab software. The approximate number of triplet repeats can be obtained by subtracting 361 bp (corresponding to the size of 5′ and 3′ flanking regions) from the PCR product length divided by 3.

### 4.3. 3′Triplet-Primed PCR (TP-PCR)

To analyze the purity of the CTG repeat tract at the 3′ end CTG repeat array, TP-PCR was performed on both strands of the CTG repeat [32,34]. The 3′ end CTG repeat was amplified using the primer downstream of the CTG repeat Somy4R-FAM (5′-FAM-CGG GTT TGG CAA AAG CAA ATT TCC CGA-3′), P3R (5′-TAC GCA TCC CAG TTT GAG ACG-3′) and P4CTG (5′-TAC GCA TCC CAG TTT GAG ACG TGC TGC TGC TGC TGC T-3′) primers as described in Tomé et al. [33]. Briefly, 20–100 ng of DNA from blood and trophoblast was amplified using 0.4 µM Somy4R-FAM and P3R primers and 0.04 µM P4CTG primer, 1× Custom master mix (Thermo Fisher Scientific, Courtaboeuf, France) and 0.06U Thermoperfect *Taq* polymerase (Peak International Products b.v, LZ Eerbeek, Netherlands)). The conditions of TP-PCR were as follows: denaturation at 94 °C for 5 min followed by 30 cycles at 94 °C for 1 min, 68 °C for 1 min 30 s and at 72 °C for 2 min and a final extension step at 72 °C for 10 min (1 cycle). The amplified product was analyzed using a 3500 XL genetic analyzer (Applied Biosystems, Foster City, CA, USA) and Gene Mapper software (Thermo Fisher Scientific, Courtaboeuf, France).

### 4.4. CTG Repeat Sequencing

CTG repeat tracts were sequenced as described in Tomé et al. [16]. Briefly, normal and expanded CTG repeat alleles were amplified by PCR using ST300F and ST300R primers and sequenced on a 3500 XL genetic analyzer (Applied Biosystems, Foster City, CA, USA). When it was necessary, purified PCR products were cloned using a TOPO-TA cloning kit (Thermo Fisher Scientific, Courtaboeuf, France) and each clone was sequenced using M13F (-20) 5′-GTA AAA CGA CGG CCA G-3′ and M13R 5′ CAG-GAA-ACA-GCT-ATG-AC-3′ primers. MacVector software was used to analyze the sequence.

### 4.5. Sequel II System from PacBio (PacBio and Sequel Are Trademarks of Pacific Biosciences)

Generation of amplicons with the CTG repeat expansion. Normal and expanded CTG repeat alleles were amplified by PCR using barcoded ST300-F and ST300-R primers (Table 4). After amplification, the PCR products for each sample were pooled and purified using the 0.5X (DM1 patients <900 CTG) or 0.45X (DM1 patients >1000 CTG) AMPure PB beads (Pacific Biosciences, Menlo Park, CA, USA) clean-up procedure. AMPure PB beads were used to remove unbound primers and the PCR product corresponding to *DMPK* normal allele. The PCR product corresponding to expanded alleles was quantified by Qubit fluorometric quantification (Thermo Fischer Scientific, Courtaboeuf, France). The quality of each purified PCR product pool was tested on an agarose gel of 1.5%.

Construction and sequencing of SMRTbell libraries. SMRTbell libraries were prepared using the SMRTbell Express Template Prep Kit 2.0, following PacBio’s “Procedure & Checklist—Preparing SMRTbell Libraries using PacBio Barcoded Universal Primers for Multiplexing Amplicons”, starting on page 11 [51]. Binding was performed with the Sequel II Binding Kit 2.1. Sequel II System run conditions included a 1-h pre-extension and 25-h movie time per SMRT Cell.

Bioinformatic analyses. Single molecule circular consensus sequences (CCS or HiFi reads) were generated from raw sequencing data using CCS version 5.0.0 (https://github.com/PacificBiosciences/ccs). Consensus reads were filtered for sequences having ≥3 passes and a minimum mean read accuracy of QV20, and sample reads were demultiplexed using lima version 2.0.1. HiFi reads were aligned to the reference using pbmm2 version 1.4.0. Repeat motifs were counted and clustered by allele using RepeatAnalysisTools (https://github.com/PacificBiosciences/apps-scripts/tree/master/RepeatAnalysisTools).

### 4.6. Haplotype Analyses

Polymorphisms flanking the CTG repeat expansion were genotyped using targeted re-sequencing developed by the Genomic Platform of the Imagine Institute. Briefly, 1 µg of genomic DNA was prepared and quantified by Qubit fluorometric quantification (Thermo Fischer Scientific, Courtaboeuf, France). In order to sequence the DM1 locus, a cosmid with a large human fragment of 45 kb containing the *DMPK* gene with 55 repeats and the flanking sequence was used to target the region of interest. The DM1 locus was sequenced using Illumina sequencing technology.

## Figures and Tables

**Table 1:** Sequel II results in family E and L2 and L3 patients carrying more than 1000 CTG repeats.

Sample	Total CCS Reads	% CCS Reads on-Target	% on-Target Reads Full-Length	Full DM1 Reads	Total Reads Analyzed	Reads < 50 CTG	Reads ≥ 50 CTG	% Expanded Allele	Estimated Repeat Size (Mode)	CCG Interruptions
E1	94,411	99.37	84	79,224	10,000	724	9276	93	5, ~447 (1099 max rpt)	No obvious interruption
E2.1	110,578	99.54	87	95,756	9999	627	9372	94	13, ~383 (831 max rpt)	2–3x CCG
E3	67,182	99.61	89	59,342	10,000	375	9625	96	31, ~173 and ~215 (478 max rpt)	6x CCG
L2	48,310	99.10	79	37,727	10,000	1291	8709	87	5, ~957 (2138 max rpt)	No obvious interruption
L3	29,512	99.02	77	22,625	9999	1670	8329	83	5, ~1156 (2081 max rpt)	No obvious interruption
A4.1	50,300	99.62	89	44,745	10,000	721	9279	93	5, ~109 (245 max rpt)	1x CAG
1201	37,407	99.44	85	31,683	9999	1813	8186	82	5, ~118 (619 max rpt)	No obvious interruption
B2	57,255	99.3	84	47,745	9995	1075	8920	89	5, ~292 (802 max rpt)	3x CCG
5289	42,349	99.31	82	34,690	9994	1595	8399	84	5, ~185 (896 max rpt)	No obvious interruption

**Table 2:** Association of myotonic dystrophy protein kinase (*DMPK*) gene-linked haplotype to intermediate 37 CTG repeat allele.

Polymorphism	RS2070736	RS572634	RS1799894	RS4646995 (*Alu* Element)	RS16939	RS527221	RS915915	CTG
Localization	Exon 1	Intron 4	Intron 5	Intron 8	Intron 9	Exon 10	Intron 11	3′UTR
G2.2	T/T	T/T	T/T	Ins/ins	G/G	C/C	T/T	5/37
G3.1	T/T	T/T	T/T	Ins/ins	G/G	C/C	T/T	16/37
DM1 haplotype A	T	T	T	Ins	G	C	T	>50

**Table 3:** Summary of DM1 interrupted alleles identified in the literature.

Sample	Sex	Ethnic Origin	CTG Size	Transmission	Pattern	Localization
**Mangin et al.**		**2021**				
G2.1	F	French	37	N/A	(CTG)6(CCGCTG)13(CTG)5	
G2.2	M	French	37	N/A	(CTG)6(CCGCTG)13(CTG)5	
G2.3	M	French	37	N/A	(CTG)6(CCGCTG)13(CTG)5	
G2.4	M	French	37	N/A	(CTG)6(CCGCTG)13(CTG)5	
G3.1	M	French	37	Paternal	(CTG)6(CCGCTG)13(CTG)5	
G3.2	F	French	37	Paternal	(CTG)6(CCGCTG)13(CTG)5	
G4.1	M	French	37	Maternal	(CTG)6(CCGCTG)13(CTG)5	
E1	M	French	546	N/A	(CTG)n	Pure
E2.1	M	French	415	Paternal (de novo)	(CTG)n(CCGCTG)(CTG)7(CCGCTG)(CTG)11	3′
E3	M	French	184/228	Paternal	(CTG)n(CCGCTG)2(CTG)3(CCGCTG)2(CTG)5(CCGCTG)(CTG)7(CCGCTG)(CTG)11	3′
**Fontana et al.**		**2020**				
I-2	F	Italian	41	N/A	(CTG)6(CCGCTG)15(CTG)5	
II-1	M	Italian	41	Maternal	(CTG)6(CCGCTG)15(CTG)5	
III-1	F	Italian	41	Paternal	(CTG)6(CCGCTG)15(CTG)5	
**Pesovic et al.**		**2018**				
DF1-1	F	Serbian	520	N/A	(CTG)n(CCGCTG)3(CTG)4(CCGCTG)2CTG(CCGCTG)(CTG)16	3′
DF1-2	M	Serbian	350	Maternal	(CTG)n(CCGCTG)3(CTG)4(CCGCTG)2CTG(CCGCTG)(CTG)16	3′
DF1-3	M	Serbian	450	Maternal	(CTG)n(CCGCTG)3(CTG)4(CCGCTG)2CTG(CCGCTG)(CTG)16	3′
DF2-1	M	Serbian	320	N/A	(CTG)n(CCG)36(CTG)n(CCGCTG)(CTG)6(CCGCTG)(CTG)11	3′
DF3-1	F	Serbian	240	N/A	CTG)n(CCG)3(CTG)6(CCG)3(CTG)6(CTG)CCG(CTG)8CCG(CTG)8	3′
DF3-2	F	Serbian	ND	Maternal	CTG)n(CCG)3(CTG)6(CCG)3(CTG)6(CTG)CCG(CTG)8CCG(CTG)8	3′
DF5-2	F	Serbian	250	Paternal (de novo)	(CTG)nCTC(CTG)26	3′
DF5-3	F	Serbian	300	Paternal	(CTG)n (sister of DF5-2)	Pure
**Cumming et al.**		**2018**				
DMGV14	F	Scotish	381	Paternal (de novo)	(CTG)180-240(CCGCTG)53-67(CTG)53-67	3′
DMGV182	M	Scotish	293	Paternal (de novo)	(CTG)200-300(CCG)(CTG)41-59	3′
DMGV15	F	Scotish	327	Paternal (de novo)	(CTG)260-320(CCGCTGCTG)10-14(CTG)15-23.	3′
**De siena et al.**		**2018**				
2 patients	N/A	Italian	N/A	N/A	(CTG)nCTC-(CCGCTG)CTG-CCG(CTG)n	5′
3 patients	N/A	Italian	N/A	N/A	(CTG)nCTC-(CCGCTG)CTG-CCG(CTG)n	3′
**Tomé et al.**		**2018**				
A1	F	French	170	N/A	(CTG)n<32(CAG)1(CTG)n	5′
A2	F	French	150	Maternal	(CTG)n<32(CAG)1(CTG)n	5′
A3	F	French	140	Maternal	(CTG)n<32(CAG)1(CTG)n	5′
A4.1	F	French	125	Maternal	(CTG)n<32(CAG)1(CTG)n	5′
A4.2	F	French	130	Maternal	(CTG)n<32(CAG)1(CTG)n	5′
A4.3	N/A	French	125	Maternal	(CTG)n<32(CAG)1(CTG)n	5′
B1	F	French	365	N/A	(CTG)11(CCGCTG)(CTG)1(CCGCTG)(CTG)3(CCGCTG)(CTG)n	5′
B2	F	French	310	Maternal	(CTG)11(CCGCTG)(CTG)1(CCGCTG)(CTG)3(CCGCTG)(CTG)n	5′
B3.1	N/A	French	300	Maternal	(CTG)11(CCGCTG)(CTG)1(CCGCTG)(CTG)3(CCGCTG)(CTG)n	5′
B3.2	N/A	French	235	Maternal	(CTG)11(CCGCTG)(CTG)1(CCGCTG)(CTG)3(CCGCTG)(CTG)n	5′
B3.3	N/A	French	250	Maternal	(CTG)11(CCGCTG)(CTG)1(CCGCTG)(CTG)3(CCGCTG)(CTG)n	5′
**Botta et al.**		**2017**				
A1	M	Italian	1000–1400	N/A	(CTG)880-1280(CTG)2(CCGCTG)(CTG)111(CCGCTG)(CTG)3	3′
A2	F	Italian	475–640	Paternal	(CTG)437-602(CTG)14(CCGCTG)(CTG)17(CCGCTG)(CTG)3	3′
A3	N/A	Italian	500	Maternal	(CTG)380(CTG)28(CCGCTG)(CTG)39(CTC)(CTG)36(CCGCTG)(CTG)7(CCGCTG)(CTG)3	3′
B1	F	Italian	740–930	N/A	(CTG)699-889(CCGCTG)2(CCG)2(CTG)3(CCGCTG)3(CTG)26	3′
B2	F	Italian	450–550	Maternal	(CTG)372-472(CTG)16(CCGCTG)(CTG)(CCGCTG)4(CTG)(CCGCTG)4(CCG)(CCGCTG)4(CCG)(CCGCTG)5(CTG)22	3′
C1	F	Italian	140	N/A	(CTG)30(CCG)2(CTG)2(CCGCTG)(CTG)104	5′
C2	F	Italian	121	Maternal	(CTG)28(CCG)2(CTG)2(CCGCTG)(CTG)87	5′
C3	N/A	Italian	113	Maternal	(CTG)31(CCG)2(CTG)2(CCGCTG)(CTG)12(CCGCTG)(CTG)62	5′
D	F	Italian	600–700	N/A	(CTG)514-614(CTG)68(CCG)9(CTG)9	3′
E	F	Italian	500–660	N/A	(CTG)404-564(CTG)33(CCGCTG)28(CTG)7	3′
F	M	Italian	250	N/A	(CTG)208(CTG)5(CCGCTG)16(CTG)5	3′
G	M	Italian	400–580	N/A	(CTG)330-510(CTG)8(CCGCTG)17(CTG)2(CCGCTG)(CTG)24	3′
H	M	Italian	175	N/A	(CTG)133)(CTG)8(CCGCTG)(CTG)4(CCG)2(CTG)(CCG)4(CTG)2(CCG)4(CTG)(CCG)2(CTG)2(CCGCTG)(CTG)8	3′
I	M	Italian	260–722	N/A	(CTG-188-650(CTG)(CCGCTG)(CTG)(CCGCTG)(CTG)6(CCGCTG)(CTG)27(CCGCTG)(CTG)6(CCGCTG)(CTG)21	3′
**Lian et al.**		**2016**				
Individu 1	N/A	ND	520	N/A	(CTG)n(CCG)50(CTG)9(CCGCTG)(CTG)6	3′
Individu 2	N/A	ND	400–480	N/A	(CTG)21(CCGCTG)(CTG)2(CCGCTG)(CTG)2(CCGCTG)(CTG)4(CCGCTG)(CTG)2(CCGCTG)2(CTG)2(CCGCTGCTG)2(CCGCTG)(CTG)4(CCGCTG)(CTG)n	5′
**Santoro et al.**		**2015**				
Pt58	N/A	Italian	118	Maternal	(CTG)32(CCGCTG)(CTG)(CCGCTG)(CTG)5(CCG)2(CTG)2(CCGCTG)(CTG)4(CCGCTG)(CTG)24(CTG)31(CCG)2(CTG)2(CCGCTG)(CTG)21(CCGCTG)(CTG)33(CCG)2(CTG)2CCGCTC(CTG)4	3′
Pt59	N/A	Italian	400–580	Paternal	(CCGCTG)7TCGCTG(CCGCTG)7(CTG)20	3′
Pt60	N/A	Italian	450–550	Maternal	(CTG)16(CCGCTG)(CTG)1(CCGCTG)4CTG[(CCGCTG)4CCG]2(CCGCTG)5(CTG)22	3′
Pt61	N/A	Italian	475–640	Paternal	(CTG)2 G (CTG)9 G (CTG)20(CTG)23TTG(CTG)4	3′
Pt62	N/A	Italian	550–700	Paternal	(CTG)5(CCGCTGCTG)46	3′
Pt63	N/A	Italian	600–700	Paternal	(CTG)68(CCGCTG)(CTG)8	3′
Pt64	N/A	Italian	600–830	Paternal	(CTG)9(CCGCTGCTG)61	3′
Pt65	N/A	Italian	740–930	Paternal	(CCGCTG)2(CCG)2(CTG)3(CCGCTG)3(CTG)7(CCGCTG)18	3′
Pt66	N/A	Italian	970	Maternal	(CTG)12(CCGCTG)(CTG)5(CCGCTG)(CTG)4(CCGCTG)(CTG)4	3′
**Santoro et al.**		**2013**				
pt1/pt62	N/A	Italian	550–700	Paternal	(CTG)2(CCGCTGCTG)5(CCG)(CCGCTGCTG)46(CTG)5	3′
pt2/pt64	N/A	Italian	600–830	Paternal	(CTG)(CCGCTGCTG)4(CCG)(CCGCTGCTG)61(CTG)9	3′
pt3	N/A	Italian	65	N/A	(CTG)(CTG/CCG)(CTG)2(CTG/CCG)(CCGCTGCTG)5(CTG)3	3′
pt4	N/A	Italian	900	N/A	(CTG)16(CCGCTG)(CTG)3(CCGCTG)(CTG)7(CCGCTG)(CTG)4(CCGCTG)(CTG)(CCGCTG)(CTG)(CCGCTG)(CTG)7	3′
pt5/pt66	N/A	Italian	970	Maternal	(CTG)12(CCGCTG)(CTG)5(CCGCTG)(CTG)4(CCGCTG)(CTG)4	3′
**Addis et al.**		**2012**				
AA	N/A	Sardinian	N/A	N/A	(CTG)3(CCGCTG)(CTG)2(CCG)CT en 3′	3′
**Radvansky et al.**		**2011**				
Sample 7	N/A	Czech	N/A	N/A	(CTG)n(CCG)38(CTG)22	3′
Sample 8	N/A	Czech	N/A	N/A	(CTG)nCTC(CTG)9(CCGCTG)2(CTG)2(CCGCTG)(CTG)4(CCGCTG)(CTG)12	3′
Sample 9	N/A	Czech	43	N/A	(CTG)6(CCGCTG)16(CTG)5	
Sample 10	N/A	Czech	43	N/A	(CTG)6(CCGCTG)16(CTG)5	
Sample 11	N/A	Czech	39	N/A	(CTG)6(CCGCTG)13(CTG)5	
Sample 12	N/A	Czech	41	N/A	(CTG)6(CCGCTG)15(CTG)5	
**Musova et al.**		**2009**				
A-1	N/A	Czech	230	Maternal	(CTG)n(CTC)(CTG)9(CCGCTG)2(CTG)2(CCGCTG)(CTG)4(CCGCTG)(CTG)12	3′
A-2	F	Czech	300	Paternal	(CTG)n(CTC)(CTG)9(CCGCTG)2(CTG)2(CCGCTG)(CTG)4(CCGCTG)(CTG)12	3′
A-3	F	Czech	400–500	Paternal	(CTG)n(CTC)(CTG)7(CCGCTG)(CTG)4(CCGCTG)(CTG)4(CCGCTG)(CTG)12	3′
A-4	M	Czech	600–800	N/A	(CTG)n(CTC)(CTG)9(CCGCTG)(CTG)4(CCGCTG)(CTG)4(CCGCTG)(CTG)12	3′
A-5	F	Czech	450–650	N/A	(CTG)n(CTC)(CTG)9(CCGCTGCTG)6(CTG)8	3′
A-6	F	Czech	650–750	Maternal	(CTG)n(CTC)(CTG)9(CCGCTGCTG)5(CTG)8	3′
A-7	M	Czech	270	Maternal	(CTG)n(CTC)(CTG)9(CCGCTGCTG)6(CTG)8	3′
B-1	M	Czech	450	N/A	(CTG)n(CCGCTG)39(CCG)(CCGCTG)3(CTG)18	3′
B-2	M	Czech	400	Paternal	(CTG)n(CCGCTG)37(CCG)12(CTG)(CCGCTG)(CTG)10	3′
C	F	Czech	700	N/A	(CTG)n(CCGCTG)2(CCG)8CTG(CCG)6CTG(CCG)6(CTG)(CCGCTG)(CCG)(CCGCTG)4(CCG)(CCGCTG)4(CTG)(CCGCTG)4(CTG)(CCGCTG)4(CCG)(CCGCTG)3(CTG)3(CCGCTG)2(CTG)10	3′
D	M	Czech	37	N/A	(CTG)6(CCGCTG)13(CTG)5	
E-1	M	Czech	43	Paternal	(CTG)6(CCGCTG)16(CTG)5	
E-2	M	Czech	43	N/A	(CTG)6(CCGCTG)16(CTG)5	
**Leeflang et al.**		**1995**				
5048	N/A	Caucasian	37	N/A	(CTG)4(CCGCTG)16(CTG)1 Alu Haplotype	
**Braida et al.**		**2010**				
III-9	M	Dutch	225/DM1-charcot	N/A	mutant allele: (CTG)n(GGC)3G(CCG)20(CCGCTG)14(CTG)35 and normal allele: (CTG)5(CCGCTG)14(CTG)	3′
IV-11	F	Dutch	38/Normal	Paternal	(CTG)5(CCGCTG)14(CTG)	
IV-12	M	Dutch	38/Normal	Paternal	(CTG)5(CCGCTG)14(CTG)	
DM1-Charcot family	N/A	Dutch	500<CTG>200	N/A	(CTG)n(GGC)3G(CCG)20(CCGCTG)14(CTG)35	3′
DM1-UC1	F	French	N/A	N/A	(CTG)n(CNG)(CTG)n(CNG)(CCGCTG)17(CTG)15	3′
DM1-UC2	M	French	N/A	Maternal	(CTG)222(CCG)5(CTG)5(CCG)5(CTG)5(CCG)5(CCGCTG)23(CTG)14	3′
DM1-UC3	N/A	French	N/A	N/A	(CTG)425(CCGCTG)4(CTG)2(CCGCTG)4(CTG)2(CCGCTG)4(CTG)4(CCGCTG)(CTG)4(CCGCTG)(CTG)4(CCGCTG)(CTG)14	3′
DM1-UC4	N/A	French	N/A	N/A	(CTG)318(CCGCTG)19(CTG)13	3′
DM1-UC5	N/A	French	N/A	N/A	(CTG)412(CCG)5(CTG)5(CCGCTG)(CCG)5(CTG)5(CCGCTG)3(CTG)4(CCGCTG)3(CTG)4(CCGCTG)3(CTG)4(CCGCTG)(CTG)5(CCGCTG)(CTG)5(CCGCTG)(CTG)5	3′
DM1-UC6	N/A	French	N/A	N/A	(CTG)516(CCG)3(CTG)(CCG)3(CTG)(CCG)2(CTG)5(CCGCTG)(CTG)5(CCGCTG)(CTG)5(CCG)3(CTG)6(CCG)3(CTG)6(CCG)3(CTG)6(CCG)3(CTG)6(CTG)26	3′
DM1-UC7	N/A	French	41	N/A	(CTG)6(CCGCTG)15(CTG)5	
DM1-UC8	N/A	French	N/A	N/A	(CTG)225(CCGCTG)(CTG)7(CCGCTG)(CTG)7(CCG)3(CTG)8(CCG)3(CTG)8(CCG)3(CTG)8(CCG)3(CTG)8(CTG)2(CCGCTG)(CTG)8	3′
DM1-UC9	N/A	French	396	N/A	(CTG)n pure et délétion 10bp en 3′	N/A
DM1-UC10	M	French	N/A	N/A	(CTG)166(CCGCTG)31(CTG)58	3′
DM1-UC11	N/A	French	N/A	Paternal	(CTG)105(CCGCTG)119(CTG)8	3′
**Leferink et al.**		**2019**				
USN04034	N/A	Dutch	>150	N/A	(CTG)n(CCGCTG)~100(CTG)n	3′
USN08692	N/A	Dutch	>150	N/A	(CTG)n(CCGCTG)114(CTG)n	3′
USN00144	N/A	Dutch	174	N/A	(CTG)24(CCGCTG)45(CTG)60	5′
USN01084	N/A	Dutch	>150	N/A	(CTG)n(CCGCTG)167(CTG)n	3′
USN01299	N/A	Dutch	>150	N/A	(CTG)n(CCGCTG)37(CTG)n	3′
**Ballester-Lopez et al.**		**2020**				
Patient 1	F	Spanish	319	N/A	(CTG)n(CCGCTG)(CTG)16(CCGCTG)(CTG)n	N/A
Patient 2	F	Spanish	241	N/A	(CTG)n(CCGCTG)(CTG)8(CCG)(CCGCTG)(CTG)(CCGCTG)(CTG)3(CCGCTG)(CTG)n	N/A
Patient 3	F	Spanish	368	N/A	(CTG)n(CCGCTG)3(CTG)3(CCGCTG)3(CTG)3(CCGCTG)2(CTG)n	N/A
Patient 4	M	Spanish	222	Maternal	(CTG)n(CCGCTG)(CTG)8(CCG)(CCGCTG)(CTG)(CCGCTG)(CTG)3(CCGCTG)(CTG)n	N/A
Patient 5	F	Spanish	547	Maternal	(CTG)n(CCGCTG)3(CTG)(CCG)2(CCGCTG)2(CTG)3(CCGCTG)2(CTG)n	N/A

**Table 4:** Primers used for long-read sequencing.

Patients	Age (year)	Interruptions	PCR Product (bp)	5′ Modification	Buffer Sequence	Barcode Name	Barcode	Primer ST-barcoded-300-F	Primer ST-barcoded-300-R
E1	56	Yes (CCG)	2551	Phosphate	GGTAG	BC1018_Forward	TCACGTGCTCACTGTG	GGTAGTCACGTGCTCACTGTGGAACTGTCTTCGACTCCGGG	GGTAGTCACGTGCTCACTGTGGCACTTTGCGAACCAACGAT
E2.1	29	Yes (CCG)	1864	Phosphate	GGTAG	BC1019_Forward	ACACACTCTATCAGAT	GGTAGACACACTCTATCAGATGAACTGTCTTCGACTCCGGG	GGTAGACACACTCTATCAGATGCACTTTGCGAACCAACGAT
E3	0	Yes (CCG)	1150	Phosphate	GGTAG	BC1020_Forward	CACGACACGACGATGT	GGTAGCACGACACGACGATGTGAACTGTCTTCGACTCCGGG	GGTAGCACGACACGACGATGTGCACTTTGCGAACCAACGAT
L2	31	No	>5000	Phosphate	GGTAG	BC1012_Forward	ACACTAGATCGCGTGT	GGTAGACACTAGATCGCGTGTGAACTGTCTTCGACTCCGGG	GGTAGACACTAGATCGCGTGTGCACTTTGCGAACCAACGAT
L3	26	No	>5000	Phosphate	GGTAG	BC1013_Forward	CTCTCGCATACGCGAG	GGTAGCTCTCGCATACGCGAGGAACTGTCTTCGACTCCGGG	GGTAGCTCTCGCATACGCGAGGCACTTTGCGAACCAACGAT
A4.1	32	Yes (CAG)	760	Phosphate	GGTAG	BC1016_Forward	CATAGAGAGATAGTAT	GGTAGCATAGAGAGATAGTATGAACTGTCTTCGACTCCGGG	GGTAGCATAGAGAGATAGTATGCACTTTGCGAACCAACGAT
1201	32	No	796	Phosphate	GGTAG	BC1017_Forward	CACACGCGCGCTATAT	GGTAGCACACGCGCGCTATATGAACTGTCTTCGACTCCGGG	GGTAGCACACGCGCGCTATATGCACTTTGCGAACCAACGAT
B2	33	Yes (CCG)	1291	Phosphate	GGTAG	BC1014_Forward	CTCACTACGCGCGCGT	GGTAGCTCACTACGCGCGCGTGAACTGTCTTCGACTCCGGG	GGTAGCTCACTACGCGCGCGTGCACTTTGCGAACCAACGAT
5289	37	No	1141	Phosphate	GGTAG	BC1015_Forward	CGCATGACACGTGTGT	GGTAGCGCATGACACGTGTGTGAACTGTCTTCGACTCCGGG	GGTAGCGCATGACACGTGTGTGCACTTTGCGAACCAACGAT

## Data Availability

All the data necessary to evaluate the conclusions in this study are present in the article. Additional data related to this paper may be requested from the authors.
